# Stress among medical students during clinical courses: a longitudinal study using contextual activity sampling system

**DOI:** 10.5116/ijme.5c94.9391

**Published:** 2019-04-02

**Authors:** Tomas Bexelius, Hanna Lachmann, Hans Järnbert-Pettersson, Susanne Kalén, Riitta Möller, Sari Ponzer

**Affiliations:** 1 Department of Medical Epidemiology and Biostatistics, Karolinska Institutet, Stockholm, Sweden; 2 Department of Health Sciences. The Swedish Red Cross University College, Huddinge, Sweden; 3 Department of Clinical Science and Education, Södersjukhuset, Karolinska Institutet, Stockholm, Sweden

**Keywords:** Medical students, stress, CanMEDS, contextual activity sampling system, CASS

## Abstract

**Objectives:**

To investigate medical students’ experiences of stress and other emotions related to their professional roles, as defined by the CanMEDS framework, by using the Contextual Activity Sampling System (CASS).

**Methods:**

Ninety-eight medical students agreed to participate of whom 74 completed this longitudinal cohort study. Data was collected between 6th and 8th term via CASS methodology: A questionnaire was e-mailed to the participants every 3rd week(21questionnaires/measurements) during clinical rotations and scientific project work term. Emotions were measured by a 7-point Likert scale (e.g., maximum stress = 7). Answers were registered through mobile technology. We used a linear mixed-model regression approach to study the association between stress over time in relation to socio-demographic and learning activities related to CanMEDS roles.

**Results:**

Participants completed 1390 questionnaires. Mean stress level over all time points was 3.6. Stress was reported as highest during the scientific project term. Learning activities related to ‘Communicator,’ ‘Collaborator,’ ‘Scholar,’ ‘Manager’ and ‘Professional’ were associated with increased stress, e.g. ’Scholar’ increased stress with 0.5 points (t_(1339)_=3.91, p<0.001). A reduced level of stress was associated with ’Health Advocate’ of 0.39 points (t_(1338)_=-2.15, p=0.03). No association between perceived stress and demographic factors, such as gender or age was found.

**Conclusions:**

An association between different learning activities related to CanMEDS Roles and feelings of stress were noted. The CASS methodology was found to be useful when observing learning experiences and might support educational development by identifying course activities linked to stress.

## Introduction

Student-activating teaching methods support learning^1^ but a student’s progress is often impacted by cognitive and emotional factors unique to the individual.^2^ Positive emotions such as interest, enthusiasm, and determination, as well as the negative emotions of irritability, nervousness and anxiety related to academics have been shown to impact learning outcomes.^3^ In addition, stress is an important independent factor affecting learning.^4^^-^^6^ Experience of stress has been shown to have a negative impact on skills-training as reported by a study done on medical students in which objective measurements such as cortisol level and blood pressure were used.^6^ Earlier studies have highlighted that stress amongst physicians starts as early as medical school and persist throughout a physician’s career.^7^ Furthermore, depressive disorders are more common among medical trainees compared to age-matched controls.^8^

All learning activities including lectures, clinical rotations and scientific projects are intended to contribute to the medical students’ professional development.  Professional roles within the medical field have been defined for example by The Royal College of Physicians and Surgeons of Canada in terms of a competency-based framework known as CanMEDS.^9^ CanMEDS is a useful tool for studying medical students’ professional development and has also been used in a Swedish context.^10^^,^^11^ However, as far as we know the association between learning activities related to CanMEDS Roles and individual perception of stress has not been studied previously from a longer perspective i.e. by following a student group through several terms. One reason might be that collecting data on students’ learning activities prospectively over a period of time can be challenging, especially if the data are intended to mirror the current state and are collected in real-time. A validated way for prospective data collection is to use the Contextual Activity Sampling System (CASS),^12^ a methodology inspired by the Experience Sampling Method,^13^ thus designed to collect data on the experience of ongoing activities by frequent distribution of questionnaires via mobile data technology. The advantage of CASS is that subjects are not affected by recall bias regarding their experiences, in contrast to summative course evaluations at the end of each rotation, i.e., conventional retrospective evaluations.^12^  Therefore, this study aimed to prospectively investigate medical students’ perceptions of stress and other emotions related to background factors and learning activities as defined by professional roles using CanMEDS framework.

**Table 1:** Socio-demographic variables at baseline among participants (N=74)

Variable		Respondents n	%
Gender			
	Male	25	34
	Female	49	66
Age group			
	<27 years	38	51
	27–30 years	18	24.5
	>30 years	18	24.5
Presence of having children			
	No	65	88
	Yes	9	12
Previous university degree			
	No	60	81
	Yes	13	18
	Missing	1	1
Family origin			
	Swedish	50	68
	European	15	20
	Non-European	8	11
	Missing	1	1

## Methods

### Educational contents and setting

Our study was carried out at a Swedish medical university with a 5.5-year-long medical education curriculum. A breakdown of the curriculum is as follows: Year 1-2 includes a study of basic sciences (e.g., cell biology, anatomy, and physiology) with short clinical placements in primary care; Year 3-5 includes clinical education (e.g., internal medicine and surgery including a total of 23 weeks of electives). Three threads (professionalism, primary care, and scientific education) run throughout the program.^14^ Our study followed the students during their terms 6-8 (year 3-4). Throughout the period of the study all participating students completed the following courses: Internal Medicine (6th term); Scientific research project (7th term); Surgery including Anesthesiology (8th term) including a 2-week inter-professional clinical rotation in a hospital ward or in the emergency room setting,^15^^,^^16^ and an integrated clinical rotation in Neurology, Psychiatry, Ophthalmology and Otolaryngology. Objective evaluations for each clinical term were comprised of a summative written examination and a practical examination, either in the form of a sit-in or an Objective Structured Clinical Examination (OSCE). The scientific research project, which equates to 30 European Credit Transfer System (ECTS) points was implemented nationwide in Sweden in 2007 as a part of harmonization to the Bologna Process and is mandatory for all medical students. The objective assessment for this was based on a written report (student thesis), an oral presentation and critical appraisal of another student’s report.

**Table 2:** Reported CanMEDS roles related to students’ ongoing learning activities

	Variable	N	%
	Total number of distributed questionnaires	1,554	100
	Total number of returned questionnaires	1,417	92
	Excluded questionnaires	27	2
	Valid questionnaires	1,390	89
			
	Number of questionnaires per student, mean (range) of valid questionnaires	18.8 (9–21)	
CanMEDS roles^*^			
	Medical expert	814	59
	Communicator	398	29
	Collaborator	260	19
	Manager	234	17
	Scholar	844	61
	Health advocate	82	6
	Professional	261	19
Term			
	6^th^	413	30
	7^th^	480	35
	8–9^th^	490	35
	Missing data/unknown	7	1

*Students were asked to report two CanMEDS roles each time. Thus, the percentages add up to >100%.

### Participants

Ninety-eight medical students in their 6th term agreed to participate in the study. To be included in the analysis each student must have completed a minimum of two questionnaires per term (6-8) in addition to the first and last questionnaires (a minimum of four responses). Moreover, the extent of missing data for each individual’s response was tolerated up to the extent of <20%. Seventy-four students met the inclusion criteria, rendering an inclusion/response rate of 76% since 24 individuals were dropped due to incomplete data. The demographics of the participants are outlined in Table 1. Females constituted 66% of the study sample, which is in line with the current ratio of women in the medical program. The mean age of the participants was 28.4 years, 12% had children, 18% had a previous university degree and 68% were of Swedish origin. As displayed in Table 2, the 74 participants together completed 1417 (91.8%) questionnaires out of 1554 (100%) possible, 27 (2%) questionnaires were excluded due to missing data, and 1390 (89%) remained for analysis. On average, the respondents answered 18.8 questionnaires each (of the 21 distributed) during the three terms 6-8.

**Table 3:** Association between stress and factors that might influence stress among 74 students measured at 21 different time points, every third week (estimates of mean difference compared with a reference or for one-point increase for the continuous variables, confidence interval (CI) from linear mixed regression analysis)

Independent Factor	Category	Adjusted for Time^1^	p-value^2^			Final Model^3^		p-value
		Estimate (95% CI)				Estimate (95% CI)		
Intercept						3.47 (2.89-4.05)		<0.001
Time of data collection^a^	Continuous	0.08 (0.03-0.12)	0.002			–0.03 (-0.09-0.02)		0.23
Squared time^a^	Continuous	–0.003 (-0.01-–0.00)	0.004			0.002 (-0.00–0.01)		0.08
Gender	Male	Reference				Reference		
	Female	0.41 (-0.02–0.86)	0.06			0.36 (-0.08–0.8)		*0.11*
Age group	<27 years	0.12 (-0.43-0.66)	0.91			Not available		
	27–30 years	0.11 (-0.52–0.74)						
	>30 years	Reference						
Presence of children	No	Reference	0.88			Not available		
	Yes	0.05 (-0.59–0.69)						
Previous university degree	No	Reference						
	Yes	–0.14 (-0.72–0.44)	0.23			Not available		
	Missing							
Family origin	Swedish	Reference	0.95			Not available		
	European	0.11 (-0.62–0.84)						
	Non-European	0.12 (-0.72–0.96)						
	Missing	N/A	-					
CanMEDS roles^b^	Total							
	Medical expert	–0.64 (-0.82–0.45)	**<0.001**			–0.16, (-0.43–0.12)		0.27
	All other roles	Reference				Reference		
	Communicator	0.17 (0.00–0.35)	*0.06*			0.40 (0.15–0.65)		0.002
	All other roles	Reference				Reference		
	Collaborator	0.02 (-0.22–0.18	*0.04*			0.43 (0.17–0.70)		0.002
	All other roles	Reference				Reference		
	Scholar	0.35 (0.18–0.52)	**<0.001**			0.51 (0.25–0.76)		<0.001
	All other roles	Reference				Reference		
	Manager	0.63 (0.41–0.84)	**<0.001**			0.72 (0.44–1.00)		<0.001
	All other roles	Reference				Reference		
	Health advocate	–0.56 (-0.90-–0.22)	*0.001*			–0.39 (-0.75–0.03)		0.03
	All other roles	Reference				Reference		
	Professional	0.03 (-0.18–0.24)	*0.08*			0.33 (0.07–0.59)		0.01
	All other roles	Reference				Reference		
Important life event^c^	Yes	0.40 (0.19–0.61)	**<0.001**			0.37 (0.16–0.58)		<0.001
	No	Reference				Reference		

1) Adjusted for a random intercept, time, squared time and each of the listed factors (one at a time). Estimates for time and squared time are reported when a random intercept, but no other factors, are included. 2) p-value is reported as a fixed effect test, and all factors with p <0.1 were included in the model adjusted for all factors, indicated in italics. 3) Adjusted for a random intercept, time-dependent factor, gender, all CanMEDS roles, and life events. The mean stress for the final estimated model was calculated as: Stress = 3.47 - 0.03*Time + 0.002*Time*Time + 0.36*Gender – 0.16 * Medical Expert + …+ 0.08*Professionals + 0.37 *Life Event, where Time = 0,1,…,21, and all other factors are 1 or 0 (reference). *Estimates of mean difference compared with a reference or for one-point increase for the continuous variables, presented with confidence interval [CI] a) Time was used a continuous variable (coded as 0,1,…,20) where each step corresponds to 3 weeks. Squared time is included to account for a changing effect of time. b) The total number of learning activities is based on all individuals’ accumulated responses, i.e., n = 1390, and the percentages are calculated relative to this number. Each CanMEDS role was treated as an independent factor in the mixed model. c) Important life event, defined as something that had an impact on the student’s daily life during the last 3-week period

**Figure 1:**
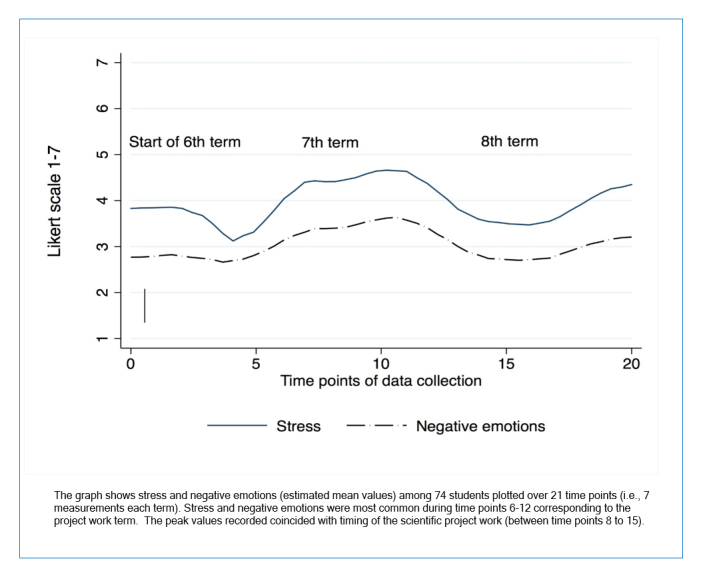
Stress and negative emotions over time

We designed this study as a longitudinal cohort study. This study is part of a larger project with previously reported results.^17^ We used the Contextual Activity Sampling System (CASS) research methodology and the CASS-query (questionnaire) to collect longitudinal data concerning ongoing learning activities and study related emotions as described in a previous paper.^17^ Measurements through questionnaires were administered via participants’ smartphones or another electronic device (personal computer, tablet, etc.) according to the CASS methodology.^18^ These were sent automatically every 3rd week to the participants via their email addresses. A text message was sent to remind the students to respond to the questionnaire within one week. After answering the questionnaire, the data were returned automatically to the database, which stored collected data and was then available as a spreadsheet for analysis. The CASS questionnaire version used in this study consisted of fifteen questions and took about 3-5 minutes to complete.

Data were collected from the autumn term of 2012 through the end of the spring term of 2014 via CASS. Students were made aware of the study on the first day of the term verbally and in writing. Through response to the initial questionnaire, students confirmed their consent to participate in writing which was an accepted method as judged by the Stockholm Regional Ethical Review Board. Each CASS questionnaire asked the students to specify the course they had been engaged in during the last three weeks. The CASS questions focused on their learning activities in relation to the CanMEDS Roles and their study related emotions during the same time period. Three types of questions were used: questions with free text answers, multiple-choice questions with stated alternatives, and ratings. The questions used in this study included background factors, learning activities related to CanMEDs Roles (multiple choice), and stress and other negative or positive emotions (ratings). The students were asked to rate their level of stress related to their studies^4^ by responding to a validated one-item question: ‘Currently, do you experience stress related to your studies?’ Further, they were asked to rate their positive (interest, enthusiasm and determination) [data not shown] and negative (nervousness, anxiety or irritability) study-related emotions on a 7-point Likert scale where 1 indicated the lowest, i.e. no stress, and 7 the highest stress score, i.e. very stressful.^17^ Additionally, with each questionnaire, students were asked to state the learning activities that had contributed most to their learning during the last 3 weeks and identify which two of the seven CanMEDS roles (Medical expert, Communicator, Collaborator, Scholar, Manager, Health Advocate and Professional) were most related to these activities.^9^ Qualitative (Yes/No) data on ‘important life event’ defined as ‘something relevant, either at work or personally, that affects your ability to study’ were also collected at each time point. For each completed questionnaire, the student was reimbursed with voucher credit that could be redeemed at a bookstore; this amounted to a maximum of 60 Euros worth if the student completed all questionnaires.

**Figure 2:**
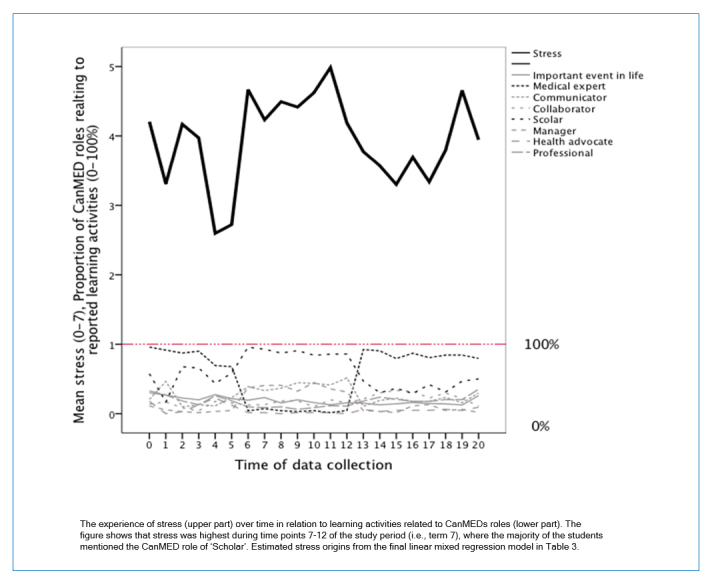
Stress level and proportion of reported learning activities over the study perioStudy design and data collection

### Data analysis and statistics

The primary outcome of this study was the reported experience of stress and negative emotions. Since stress and negative emotions showed similar results over time, we focused our results on stress (Figure 1). The potential impact of the number of terms was evaluated and divided into 6th, 7th and 8–9th terms to achieve three balanced categories in terms of relative frequency. The number of completed questionnaires was lower in the 9th term since only one questionnaire was distributed during this term, and therefore it was merged with the 8th term. We used a linear mixed-model regression approach to study the association between stress and type of CanMEDS role over time in relation to socio-demographic factors. Mixed models compensated for partially missing data and allowed us to examine time-dependent effects and repeated observations on the same individual.^19^ We used the following model strategy. First, we investigated whether the intercept of the outcome (i.e., stress) randomly varied between individuals at baseline, which was the case (i.e., a random intercept). Second, we studied the effect of time, which we regarded as a fixed effect (i.e., fixed slope). Also, ‘squared time’ was included to account for a diminished/changing effect over time. Time was coded as 0, 1….20, which implies that the intercept (time 0) is interpreted as the average stress at baseline for individuals belonging to the reference category.  A composite score of the average means of the different negative emotions was calculated over time.

Third, to study the association between stress and each of the independent factors after adjusting for time, we used a model with the random intercept, fixed time and squared time and added each factor individually. The following independent factors were evaluated: gender, age, the presence of children, family origin, previous university degree and the seven different CanMEDS role(s) analysed separately as independent variables (yes/no). These factors were tested owing to a priori hypotheses. In addition, we also included occurrences of ‘an important life event’ in the model.

Finally, to study which factors were associated with stress after adjusting for time and other relevant variables, we used a mixed model consisting of all factors that showed an association (p <0.1) with stress after adjustment for time, squared time and a random intercept. This selection method was done to choose variables to be included in the final model. The observed and predicted mean stress is presented in Figure 2. The estimated model is based on the final model with random intercept, and fixed time, time square, gender and all CanMEDS role and life event (Table 1). All statistical tests were performed two-sided and the significance level was set to p <0.05. Statistical analyses were performed using software packages SPSS version 22 and Stata version 14.

## Results

### Stress in relation to professional roles as defined by CanMEDS framework

The experience of stress and an average of the negative and positive emotions related to studies over time are shown in Figure 1. A peak level of stress was reported during the 7th term when students carried out their individual research projects. Also, the pattern of negative emotions followed that of stress with a peak in term 7 (Figure 1).

The most commonly reported CanMEDS roles the students’ learning activities were related to ‘Medical Expert’ (59%) and ‘Scholar’ (61%), as shown in Table 2.

The stress was highest during the middle of the study period (i.e., term 7) (Figure 1). Figure 2 shows the experience of stress in relation to the different CanMEDs roles and show that stress was highest during term 7 (Figure 1) where the majority of the students mentioned the role of ‘Scholar’. We present the estimated stress from the mixed model (upper part of the graph in Figure 2) for the final model in Table 3, and the proportion of individuals at each time point reporting learning activities related to the different CANMEDs roles (lower part of Figure 2).

There was a linear association between stress and time, corresponding to an 0.08 point increase in stress after every 3-week data collection point during the whole study period, but the association declined over time (quadratic time: -0.003) (Table 3). Although not significant, female gender was associated with 0.36 higher level of stress compared with male gender (t_(__73)_=1.64, p=0.11).

All 7 CanMEDS roles were associated with stress (p <0.1), and therefore included in the multivariable model (Table 3).  Learning activities related to the CanMEDS role of ‘Medical Expert’ decreased stress by 0.64 points in the univariable model (t_(__1366)_=6.81, p<0.001), but this effect did not remain significant (t_(1330)_=-1.10, p=0.27) in the multivariable model (Table 1). Interestingly the roles of ‘Communicator,’ ‘Collaborator,’ ‘Scholar,’ ‘Manager’ and ‘Professional’ were all associated with increased stress (Table 3). Conversely, the role of ‘Health Advocate’ reduced levels of stress by 0.39 points (t_(__1338)_=-2.15, p=0.03) compared with the other roles (Table 3). Finally, important life events increased stress with 0.37 points (t_(__1373)_=3.51, p<0.001).

Thus, after adjustments for time-varying factors (i.e., life events and reported CanMEDS roles) and for non-time-varying factor (gender), neither the effect of time nor gender was significant. Thus, stress was more related to time-varying factors than to a general ‘time trend.’ Other socio-demographic factors, such as age, the presence of children, previous university degree or family origin, were not associated with stress as outlined in Table 3.

## Discussion

We focused on medical students’ perceptions of stress during their clinical rotations.  Since negative emotions and stress showed a similar pattern, we chose to focus mainly on stress. The students’ stress levels were highest during their 7th term when they carried out their scientific research projects and also when they most often reported learning activities related to the CanMEDS role ‘Scholar’. The key finding was that activities related to different CanMEDS roles were more important in determining the level of stress than any socio-demographic background factors, including gender and age.

This is the first study of its kind investigating CanMEDS roles in relation to stress and negative emotions reported by medical students via Contextual Activity Sampling System, CASS. The CASS method gave us a possibility to follow students over time and by doing so get contextual data where actual learning activities and be related to emotions, and specifically feelings of stress, during studies. Using the CASS also helped us to avoid recall-bias commonly affecting retrospectively collected data.  Even if CanMEDS was originally designed for the Canadian context, it has been translated and used in many other countries including Sweden where this study was conducted. CanMEDS is also a frequently used framework for analysing outcome-based medical education.^9^ We chose to use validated and established scales for measuring stress and emotions^4^^,^^20^ to ensure that our results can be compared with other studies.

We studied the level of stress in relation to background factors such as gender, family origin, and educational background, but we did not find any statistically significant differences. This was somewhat surprising, considering that the descriptive analyses before adjustments in the mixed models showed, for example, that female gender was related to higher stress levels. None of the other background factors, such as age, the presence of children and a previous university degree, were associated with stress, contrary to our hypothesis before the study started. However, this finding is in line with a study^21^ reporting on medical students stress levels which showed that levels of anxiety and depression were higher among students compared to reference group but there were no significant differences in stress levels related to gender, migration background or employment status.

The results also showed that activities related to different CanMEDS roles were more important than any background factors. Statistically significant associations between stress and almost all CanMEDS roles were found.  The role of ‘Medical Expert’ was not associated with stress, while practicing the other roles were related to an increased stress level, except the role of ‘Health Advocate,’ which showed an association with lower stress levels. Another finding was that activities related to the roles ‘Manager’, and ‘Professional’ were associated with higher stress levels. This result could be due to students having less experience in these roles like the role of ‘Medical Expert’ dominates during clinical courses as per curriculum design even if an integrated curriculum should address all CanMEDS Roles during all terms.^21^ Notably, the experience of stress peaked during the 7th term when the students carried out their individual scientific projects. This finding was not surprising since this term differs from the others in that an individual research project is the single most substantial work students are solely responsible for in medical school.^22^ Even if the students had continuous supervision, they were still required to meet deadlines, and to independently produce a written report of acceptable academic standards based on collected data that they analyzed.^23^

This is an interesting finding since many studies on stress among medical student focus on time and other aspects of being a student but not the learning activities they are involved in. For example, in a study focusing on wellness programs for medical students, it was pointed out that stress increased during studies and peaked at 3rd year of medical school.^24^ This paper does not report the type of educational activities the students did during their 3rd year why their results are difficult to compare to ours. However, in contrast to their study, our results did not show increased stress during 3rd or 4th year, but that stress was mainly related to the type of activities the students were involved in.

### Limitations

A weakness of this study is the possible selection bias since the non-responders might have experienced more stress, which may have been a reason to not respond at all. Even so, we believe that our results have a high internal validity owing to the validated instruments, good response rate during all three terms and relatively complete data on potential confounders.  Another weakness is that a data collection over a long period of time often results in missing values, which was also the case in our study, albeit at low levels. Our response rate was over 75%, and for participants included, almost all data were complete. However, our findings need to be evaluated in other learning contexts since educational and demographic factors might differ amongst countries.

## Conclusions

In conclusion, we showed that there is an association between different learning activities related to CanMEDS roles and feelings of study related stress. Notably, stress increased during students’ scientific project term when most activities were regarded as ‘Scholar.’ The effect of a particular learning activity was more important than any background factor studied. Our study shows that CASS methodology is a reliable way of collecting data on emotions that largely correlate with that of stress. The study result may be used to monitor stress levels continuously and identifying specific course activities/learning activities where students are suffering from most stress and negative emotions.

### Acknowledgements

The authors thank the students at Karolinska Institutet who participated in the study. We also would like to acknowledge the contribution from Dr. Ajla Wasti at The Royal Marsden Hospital, Sutton UK who has proofread and language-edited the manuscript.  This study was supported by grants provided by the Stockholm County Council (ALF-project).

### Conflict of Interest

The authors declare that they have no conflict of interest.
